# Real Time Imaging of Deuterium in a Duplex Stainless Steel Microstructure by Time-of-Flight SIMS

**DOI:** 10.1038/srep19929

**Published:** 2016-02-02

**Authors:** O. Sobol, F. Straub, Th. Wirth, G. Holzlechner, Th. Boellinghaus, W. E. S. Unger

**Affiliations:** 1 BAM – Federal Institute for Materials Research and Testing, Unter den Eichen 87, D-12205 Berlin, Germany

## Abstract

For more than one century, hydrogen assisted degradation of metallic microstructures has been identified as origin for severe technical component failures but the mechanisms behind have not yet been completely understood so far. Any *in-situ* observation of hydrogen transport phenomena in microstructures will provide more details for further elucidation of these degradation mechanisms. A novel experiment is presented which is designed to elucidate the permeation behaviour of deuterium in a microstructure of duplex stainless steel (DSS). A hydrogen permeation cell within a TOF-SIMS instrument enables electrochemical charging with deuterium through the inner surface of the cell made from DSS. The outer surface of the DSS permeation cell exposed to the vacuum has been imaged by TOF-SIMS vs. increasing time of charging with subsequent chemometric treatment of image data. This *in-situ* experiment showed evidently that deuterium is permeating much faster through the ferrite phase than through the austenite phase. Moreover, a direct proof for deuterium enrichment at the austenite-ferrite interface has been found.

The popularity of Duplex Stainless Steel (DSS) arises from an attractive combination of properties, including high strength and excellent corrosion resistance. Due to these properties, this type of steel is being used in many applications and environments which frequently provide critical conditions for hydrogen absorption in metallic microstructures^1^. By cathodic charging or during free corrosion of DSS in hydrogen environments retarding recombination, overall hydrogen concentrations of more than 40 at.ppm have frequently been observed and found to be harmful^2^,^3^. Dependent on the respective solution and trapping mechanisms, hydrogen taken up from various sources might cause significant degradation of the mechanical properties and consequently might entail hydrogen assisted cracking (HAC) of components, as documented by severe failure cases. Although HAC represents an important issue in industrial applications and attracted enormous research attention for decades, the mechanisms of hydrogen assisted crack initiation and propagation as well as those of hydrogen transportation in the respective microstructures are not yet fully understood^3^,^4^,^5^,^6^,^7^. Local *in-situ* characterization of the hydrogen distribution in steel microstructures represents a key for elucidation of HAC as well as hydrogen transportation in the respective microstructures^8^,^9^. One, if not the only, laterally resolving method of chemical analysis for imaging the locally hydrogen distribution and interaction in a specific microstructure is secondary ion mass spectrometry (SIMS)^10^. Up to the present, all of the SIMS experiments aiming on the elucidation of hydrogen effects were carried out using externally charged samples and dynamic SIMS instruments (for a survey cf. SI). In contrast, real time imaging results have been obtained in the present study by static ToF-SIMS monitoring of the deuterium distribution in a DSS microstructure while continuously charged for the first time (cf. Fig. 1). In the experiments, deuterium that permeated through the steel’s microstructure has been imaged at the outer surface of an electrochemical permeation cell made from DSS itself which is located within the ultra-high vacuum chamber of the instrument (for details see SI). Analysis of that vacuum/steel interface avoids any impact of the electrolyte on the imaged surface. In the permeation experiment, the DSS surface has been investigated with increasing time of charging: First deuterium secondary ions were detected after 28 days of charging, saturation after 37 days. The limited sensitivity of SIMS (secondary ion emission probabilities may be small as 10^−4^) leads to an underestimation of the “break-through” time which is the time of charging required for detecting the first intentionally introduced deuterium on the analyzed surface. To gain a maximum of information from the SIMS image data acquired multivariate data analysis by Principal Component Analysis (PCA) has been performed. By using key co-variations between element and fragment secondary ions the image contrast between features is substantially increased. In comparison to a previous SIMS study of deuterium effusion from the DSS sample reported by the authors^11^ the steps beyond the present experiment al state are (i) the real time monitoring of the permeation, diffusion and distribution of deuterium in individual phases of the DSS microstructure and (ii) the multivariate treatment of SIMS image data.

The use of the hydrogen isotope deuterium emerges from the requirement to distinguish between hydrogen species diffused through the microstructure of DSS to the imaged surface and hydrogen containing residual gas species adsorbed thereon. Of course, there are differences in diffusibility and solubility for both hydrogen isotopes, but these range within the same order of magnitude. It is well-known that, in principal, deuterium behaves very similar to hydrogen in steel microstructures^7^,^10^,^12^,^13^,^14^.

Results of the *in-situ* permeation experiment are displayed in Fig. 2. Figure 2a shows the scores image for the first principle component (PC1) obtained from the positive secondary ion data set comprising matrix related secondary ions. The related loadings plot (cf. Figure S2 in the SI) indicate Ni^+^, Mn^+^ and Fe^+^ as key secondary ions that characterize the variance captured by PC1 obtained from the positive secondary ion data set. Thus the different phases in DSS can clearly be distinguished. The ferrite appears dark and the austenite appears bright. Figure 2b exhibits the PC1 scores image delivered by PCA of the negative secondary ion data set where deuterium related secondary ions contribute to. In this case the related loadings plot (cf. Figure S2 in the SI) indicates D^−^ and OD^−^ as key (fragment) secondary ions that characterize the variance captured by PC1. By comparison of Fig. 2a,b, a preferential deuterium accumulation in the ferrite phase becomes evident. For single grains inside austenite and ferrite phases, intensity variations in Fig. 2b have been observed. This observation is assumed to be due to the dependency of the secondary ion emission probability of deuterium related secondary ions on the respective grain lattice plane exposed to the impinging primary ions.

Based on Fig. 2a,b, a higher permeation of deuterium and faster saturation of the ferrite phase is concluded when compared to the austenite phase. This experimental result is consistent with literature data, where deuterium in the ferrite phase is reported to have an about five orders of magnitude higher diffusivity^7^,^15^,^16^. By this, the current *in-situ* measurements provide evidence to previous statements^15^,^17^,^18^ based on models and experiments that deuterium preferentially diffuses through the ferrite matrix. In accordance to the diffusion coefficient^18^, within the time frame of the reported experiment, only a minor amount of deuterium will be diffusing into and trapped in the austenite grains at considerably lower diffusion coefficients.

Reduction of the negative image data matrix used for score images displayed in Fig. 2b by the use of only a mass range 20–50 m/z reveals images shown in Fig. 2c,d. Figure 2c presents an image of the scores of PC1 where significant positive loadings have been found only for m/z = 26, i. e. CN^−^ fragment ions (cf. Figure S2 in the SI). Comparison of Fig. 2c with Fig. 2a reveals that these occur in areas corresponding to the austenite phase. Moreover, the comparison of Fig. 2c with Fig. 2b reveals that certain regions in the ferrite phase where a bright region related to CN^−^ correlates with a bright area representing high deuterium concentrations.

Figure 2d displays the PC2 scores image belonging to the PC1 image in Fig. 2c. The loadings plot for PC2 shows strong positive loading exclusively at m/z = 43 assigned to CN_2_HD^−^ (cf. Figure S2 in the SI) and displays distinct positive scores at grain boundaries within a specific phase and high positive scores at ferrite-austenite interfaces. This image provides evidence that regions at the grain boundaries and interfaces contain carbon and nitrogen as well as accumulated deuterium at the same time. Such findings indicate that grain and phase boundaries act as /sinks in the microstructure which attract hydrogen or deuterium ions^19^,^20^,^21^.

The SIMS data sets leading to images displayed in Fig. 2 have been taken from a region of interest (ROI) selected at the beginning of the *in-situ* permeation experiment which has been analyzed many times before to identify the break-through of deuterium. To rule out any unintended measurement artifacts (e.g. due to beam damage) a fresh ROI was selected for ToF-SIMS imaging after 37 days of charging (shown in Fig. 3). Again, the ferrite phase appears dark and the austenite is bright in Fig. 3a. Also, the significantly higher deuterium concentration in the ferrite grains in comparison to the austenite has been confirmed by Fig. 3b. Similar results to those obtained after break-through of deuterium have been found for the saturated state. Additionally an ion-induced secondary electron image (Fig. 3c) of the duplex microstructure at the new ROI is taken where also phase and even grain boundaries can be distinguished.

Principally the *in-situ* permeation experiment should enable also a determination of kinetic data as, e.g., overall diffusion coefficients. Unfortunately quantitative evaluation of such values will be limited by the detection limit of the SIMS instrument for deuterium and the tortuous effect occurring in a two phase alloy^17^,^18^. However, it has to be expected that most of the deuterium detected at the time of the break-through permeated predominantly through the ferrite phase on paths circumventing the austenitic phase (cf. ref. [15]) characterized by considerably lower diffusivity and higher solubility for deuterium.

It has been demonstrated for a technically relevant multiphase metallic microstructure, here DSS, that imaging ToF-SIMS together with subsequent image data treatment by PCA is a powerful approach for phase identification (positive SIMS mode) and *in-situ* visualizing the deuterium distribution (negative SIMS mode). The *in-situ* approach using a deuterium charging cell in the analysis chamber of a ToF-SIMS instrument provided clear experimental evidence for a preferred diffusion of deuterium through the ferrite phase, while *ex-situ* SIMS studies could only show preferential accumulations in the phases subsequently to charging with hydrogen or deuterium^10^,^22^.

The experiment also enables an investigation of interactions of deuterium with grain and phase boundaries, carbon and nitrogen in the DSS microstructure. However, it addresses the diffusion and permeation of deuterium through the microstructure without any external mechanical loading on the specimen. It is anticipated that, under these specific circumstances, deuterium accumulates in the lattice and is trapped at dislocations and already existing twins^3^ with much higher solubility and lower diffusivity of hydrogen/deuterium in austenite as compared to ferrite.^7^ The authors currently develop an advanced experiment aiming on the realization of an *in-situ* mechanical loading of deuterium charged specimens to study the degradation process of each phase under these more complex circumstances by imaging ToF-SIMS.

The real time and *in-situ* imaging ToF-SIMS based approach introduced in this letter widens the perspectives for experiments aiming on deuterium and hydrogen (or even other mobile constituents) behavior in a range of relevant metallic microstructures. It will contribute to a better understanding of hydrogen and deuterium permeation and subsequent cracking in metallic microstructures known to be crucial for the design of engineering components in industrial branches with high impact as petrochemical, off-shore constructions and oilfield applications.

Finally it should be mentioned that the *in-situ* ToF-SIMS experiment reported here is part of recent community overarching activities to develop new experiments e.g. on the big and flexible sample holder within the analysis chamber of ION TOF’s ToF-SIMS instruments. One example is a microfluidic device for *in situ* ToF-SIMS analysis of the Liquid/Vacuum interface introduced in ref. 23.

## Methods

### ToF-SIMS analysis procedure

ToF-SIMS measurements were performed using a ToF-SIMS IV instrument (ION-TOF GmbH, Muenster, Germany). 25 keV Bi^+^ primary ions delivered by a liquid metal ion gun (LMIG) were used for analysis. A sputter ion gun providing 3 keV Ar^+^ ions for sputtering has been used for cleaning the region of interest. Previously to the measurements an area of 300 × 300 μm^2^ was sputtered by Ar^+^ primary ions for 5 min to remove surface contaminants originating from the cell production process. ToF-SIMS data from the surface of the steel were acquired from 100 × 100 μm^2^ sample ROI performing the burst alignment mode with a beam diameter of approximately ~250 nm providing sufficient lateral resolution for imaging the steel’s micro structure. The information depth of the SIMS method equals to the first and second monolayer of the steel surface^24^.

The first step of the experiment was to image the finely polished DSS surface of the cell before charging by ToF-SIMS in positive mode. A representative ROI of 100 × 100 μm^2^ was selected to identify the austenitic and the ferritic phases by the detection of Cr^+^, Mn^+^ and Fe^+^ secondary ions; first with instrument settings delivering high mass resolution and thereafter with settings for high lateral resolution^25^. Subsequently, negative secondary ion images were acquired from one and the same ROI at increasing times of charging with deuterium over a period up to saturation (37 days).

### PCA and data preprocessing

Image data were treated by PCA (for details see SI). PCA was performed using the Solo + Mia toolbox (v7.5.2, Eigenvector Research Inc.) which were run under MATLAB (v7.9.0.529, MathWorks Inc.). Prior to principal component analysis the raw data of secondary ions were pre-processed by normalization to the total intensity of each respective mass spectrum, Poisson scaling and mean centering finally.

### Design and details of the *in-situ* charging experiment

**Figure 1:**
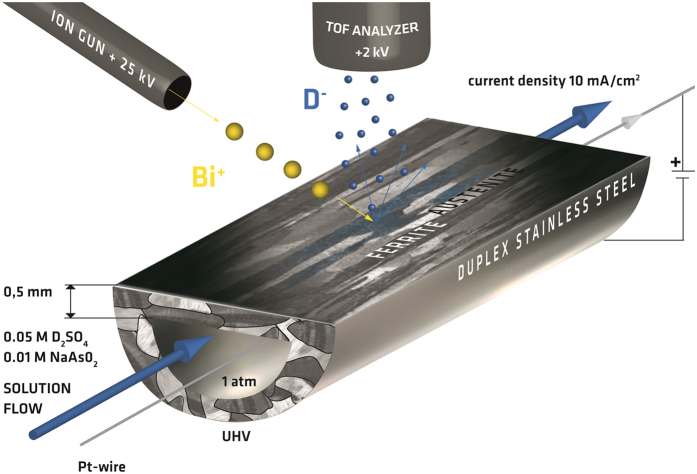
Schematic view of the unique design of the real time and *in-situ* deuterium permeation experiment within ultra-high vacuum chamber of the ToF-SIMS instrument. This set-up enables to monitor the deuterium distribution in the DSS microstructure at the polished vacuum exposed surface of the charging cell made from 2205 DSS. Deuterium becomes visible after permeation through a 0.5 mm thick DSS sheet.

The results have been obtained by a novel experimental approach, i.e. by mounting a specifically designed cell (Fig. 1) in the ultra-high vacuum (UHV) analysis chamber of the ToF-SIMS instrument. The cell was made using a rolled cylindrical bar of a duplex stainless steel grade 2205 (UNS S31803, EN 1.4462). The material was purchased from ThyssenKrupp and specified by Deutsche Edelstahlwerke GmbH according to applicable standards^26^,^27^,^28^,^29^,^30^,^31^ to have a ferrite content of 51%. After rolling, the bar was solution annealed in 1050 °C and water quenched. The cell was fully made from this DSS material. The flat outer surface of the cell exposed to UHV and the primary ion beam has been polished for *in-situ* imaging by ToF-SIMS and is illustrated in Fig. 1. The DSS material has been machined in a way enabling observation of diffusion perpendicular to the rolling direction. This cell allows continuous electrochemical charging of the duplex stainless steel (DSS) with deuterium from inside and analyzing its outer surface exposed to UHV at the same time. The inner surface of the cell was flat and plan-parallel to its monitored outer surface. In that way a 0.5 mm thick steel membrane has been established for permeation experiments with deuterium. To introduce deuterium into the steel a continuous flow of an electrolyte solution of 0.05 M D_2_SO_4_ and 0.01 M NaAsO_2_ (recombination poison) in D_2_O was realized through the tubular test sample. This was accomplished by pumping the electrolyte through a flexible polymer pipe. The pipe was lead from outside the UHV chamber to the tubular sample within the chamber by using flexible stainless steel bellows. A Pt wire was acting as counter electrode (anode) in the galvano-static charging process using a current density of 10mA·cm^−2^.

## Additional Information

**How to cite this article**: Sobol, O. *et al*. Real Time Imaging of Deuterium in a Duplex Stainless Steel Microstructure by Time-of-Flight SIMS. *Sci. Rep.* **6**, 19929; doi: 10.1038/srep19929 (2016).

## Supplementary Material

Supplementary Information

## Figures and Tables

**Figure 2:**
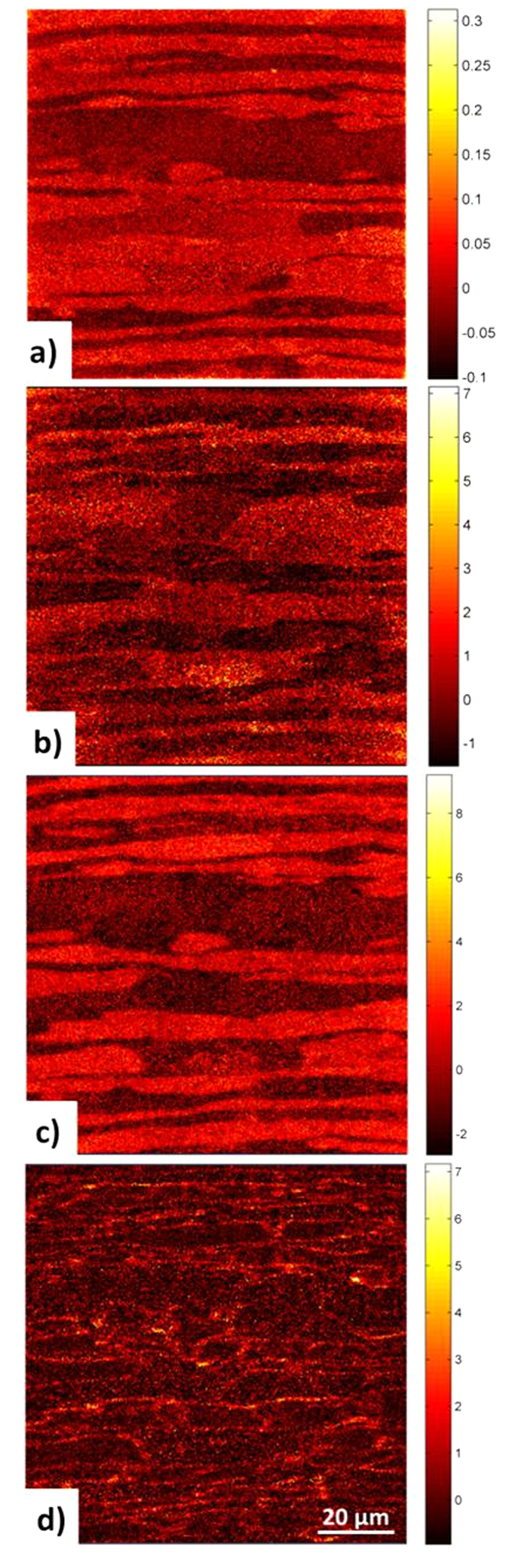
Principle component score images of the ToF-SIMS data sets acquired after 34 days of charging. (**a**) PC1 scores image for identifying phases using the positive ToF-SIMS data set acquired after 34 days of charging DSS with deuterium by using a peak list with secondary ions covering the main alloying elements. The bright regions represent the austenite phase (as proved by the loadings plot in shown Figure S2a). (**b**) PC1 scores image identifying locations rich in deuterium obtained from the negative SIMS data set acquired after 34 days and using the full peak list. Bright pixel indicate positive scores and correlate with the ferrite phase. (**c**) PC1 scores image obtained from the same data set but using a different peak list prepared to focus on carbides and nitrides. This image is identifying locations rich in N and C. Bright pixels indicate positive scores and correlate with the austenite phase. (**d**) PC2 scores image identifying locations rich in N, C and D (bright pixel means positive score) indicating graded accumulation of deuterium at the ferrite and austenite grain boundaries and at the ferrite/austenite interface (highest scores!) as well. All ToF-SIMS image acquisition settings and parameters are given in table S3 in SI.

**Figure 3:**
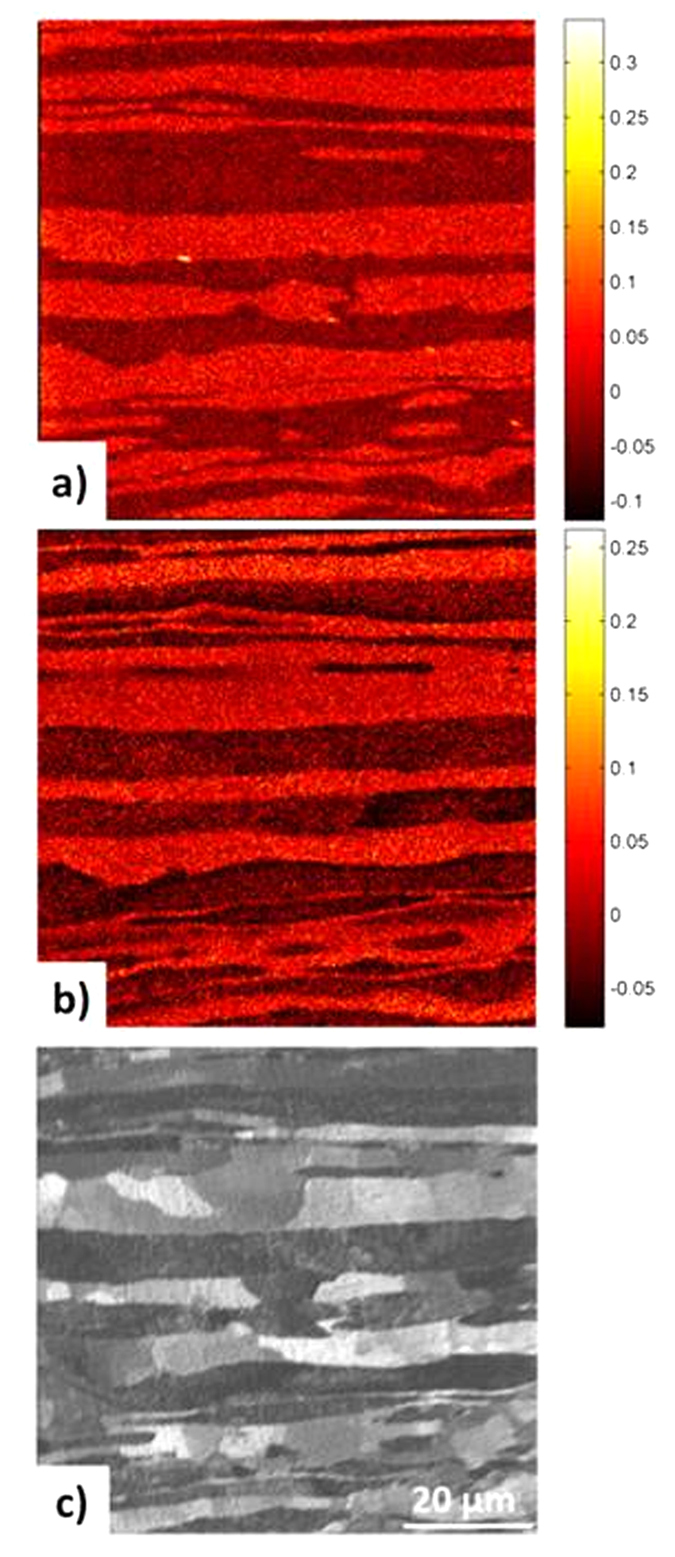
Principle component score and secondary electron images of the ToF-SIMS data sets acquired after 37 days of charging. (**a**) PC1 scores image obtained from the positive SIMS data set acquired after 37 days of charging DSS and used to identify phases in the DSS micro structure. A fresh region of interest (ROI) has been selected. The bright regions represent the austenite phase (as shown in the loading plot in Figure S2a). (**b**) PC1 scores image related to locations rich in deuterium. The image was generated by PCA of a negative ToF-SIMS image data set obtained after loading with deuterium for 37 days. The bright deuterium-rich areas have positive scores and correspond to the ferrite phase while the dark areas have negative scores and relate to austenite grains. (**c**) Ion induced secondary electron (SE) image of displaying grains in the DSS microstructure at the selected region of interest.

## References

[b1] Alvares-Armas I. Duplex Stainless Steels: Brief History and Some Recent Alloys. Recent Patents on Mechanical Engineering 1, 51–57 (2008).

[b2] Nagao A., Martin M. L., Dadfarnia M., Sofronis P. & Robertson I. M. The effect of nanosized (Ti,Mo) C precipitates on hydrogen embrittlement of tempered lath martensitic steel. Acta Materialia 74, 244–254, 10.1016/j.actamat.2014.04.051 (2014).

[b3] Robertson I. M. . Hydrogen Embrittlement Understood. Metallurgical and Materials Transactions a-Physical Metallurgy and Materials Science 46A, 2323–2341 (2015).

[b4] Dadfarnia M. . Recent advances in the study of structural materials compatibility with hydrogen. Advanced Materials 22, 1128–1135 (2010). 20217854 10.1002/adma.200904354

[b5] Song J. & Curtin W. A. Atomic mechanism and prediction of hydrogen embrittlement in iron. Nature Materials 12, 145–151, 10.1038/nmat3479 (2013). 23142843

[b6] Seita M., Hanson J. P., Gradečak S. & Demkowicz M. J. The dual role of coherent twin boundaries in hydrogen embrittlement. Nature communications 6, 6, 10.1038/ncomms7164 (2015). 25652438

[b7] Marchi C. S., Somerday B. P. & Robinson S. L. Permeability, solubility and diffusivity of hydrogen isotopes in stainless steels at high gas pressures. International Journal of Hydrogen Energy 32, 100–116, 10.1016/j.ijhydene.2006.05.008 (2007).

[b8] Robertson I. M. The effect of hydrogen on dislocation dynamics. Engineering Fracture Mechanics 68, 671–692, 10.1016/s0013-7944(01)00011-x (2001).

[b9] Barnoush A., Zamanzade M. & Vehoff H. Direct observation of hydrogen-enhanced plasticity in super duplex stainless steel by means of *in situ* electrochemical methods. Scr. Mater. 62, 242–245, 10.1016/j.scriptamat.2009.11.007 (2010).

[b10] Oltra R., Bouillot C. & Magnin T. Localized hydrogen cracking in the austenitic phase of a duplex stainless steel. Scr. Mater. 35, 1101–1105, 10.1016/1359-6462(96)00293-x (1996).

[b11] Straub F., Boellinghaus T., Unger W. E. S. & Mente T. In International Hydrogen Conference (IHC 2012). (eds Somerday B. & Sofronis P.) 505–513 (ASME press, 2014).

[b12] Fukai Y. The Metal-Hydrogen System-Basic Bulk Properties. Vol. 261 (1993).

[b13] Katsuta H. & Furukawa K. Hydrogen and Deuterium Transport through Type-304 Stainless-Steel at Elevated-Temperatures. Journal of Nuclear Science and Technology 18, 143–151, 10.3327/jnst.18.143 (1981).

[b14] Quick N. R. & Johnson H. H. Permeation and Diffusion of Hydrogen and Deuterium in 310 Stainless-Steel, 472-K to 779-K. Metallurgical Transactions a-Physical Metallurgy and Materials Science 10, 67–70, 10.1007/bf02686408 (1979).

[b15] Mente T. & Boellinghaus T. Modeling of Hydrogen Distribution in a Duplex Stainless Steel. Weld. World 56, 66–78 (2012).

[b16] Olden V., Thaulow C. & Johnsen R. Modelling of hydrogen diffusion and hydrogen induced cracking in supermartensitic and duplex stainless steels. Mater. Des. 29, 1934–1948, 10.1016/j.matdes.2008.04.026 (2008).

[b17] Turnbull A. & Hutchings R. B. Analysis of Hydrogen-Atom Transport in a 2-Phase Alloy. Materials Science and Engineering a-Structural Materials Properties Microstructure and Processing 177, 161–171, 10.1016/0921-5093(94)90488-x (1994).

[b18] Owczarek E. & Zakroczymski T. Hydrogen transport in a duplex stainless steel. Acta Materialia 48, 3059–3070, 10.1016/s1359-6454(00)00122-1 (2000).

[b19] Ichitani K. & Kanno M. Visualization of hydrogen diffusion in steels by high sensitivity hydrogen microprint technique. Science and Technology of Advanced Materials 4, 545–551 (2003).

[b20] Au J. & Birnbaum H. K. On the Formation of Interstitial-Hydrogen Clusters in Iron. Scripta Metallurgica 15, 941–943, 10.1016/0036-9748(81)90283-0 (1981).

[b21] Hannula S. P., Hanninen H. & Tahtinen S. Influence of Nitrogen Alloying on Hydrogen Embrittlement in Aisi 304-Type Stainless-Steels. Metallurgical Transactions a-Physical Metallurgy and Materials Science 15, 2205–2211, 10.1007/Bf02647103 (1984).

[b22] Tanaka T., Kawakami K. & Hayashi S.-I. Visualization of deuterium flux and grain boundary diffusion in duplex stainless steel and Fe-30% Ni alloy, using secondary ion mass spectrometry equipped with a Ga focused ion beam. Journal of Materials Science 49, 3928–3935, 10.1007/s10853-013-7956-7 (2014).

[b23] Yang L., Zhu Z. H., Yu X. Y., Thevuthasan S. & Cowin J. P. Performance of a microfluidic device for *in situ* ToF-SIMS analysis of selected organic molecules at aqueous surfaces. Anal Methods-Uk 5, 2515–2522, 10.1039/c3ay26513g (2013).

[b24] Benninghoven A. Surface analysis by secondary ion mass spectrometry (SIMS). Surf. Sci. 299, 246–260 (1994).

[b25] Straub F. . Imaging the microstructure of duplex stainless steel samples with TOF-SIMS. Surface and Interface Analysis 42, 739–742, 10.1002/sia.3385 (2010).

[b26] ASTM A182/A182M, Standard Specification for Forged or Rolled Alloy and Stainless Steel Pipe Flanges, Forged Fittings, and Valves and Parts for High-Temperature Service. (ASTM International, West Conshohocken, PA, 2010).

[b27] ASTM A276, Standard Specification for Stainless Steel Bars and Shapes. (ASTM International, West Conshohocken, PA, 2010).

[b28] ASTM A479/A479M, Standard Specification for Stainless Steel Bars and Shapes for Use in Boilers and Other Pressure Vessels. (ASTM International, West Conshohocken, PA, 2010).

[b29] DIN EN 10088, Stainless steels-Part 5: Technical delivery conditions for bars, rods, wire, sections and bright products of corrosion resisting steels for construction purposes. (European Committee for Standardization (CEN), 2005).

[b30] DIN EN 10272, Stainless steel bars for pressure purposes. (German Institute for Standardization, 2007).

[b31] NACE MR0175/ISO 15156-3, “Petroleum and Natural Gas Industries—Materials for Use in H_2_S-Containing Environments in Oil and Gas Production”. (NACE International, Huston, TX, 2003).

