# Supplementary material for: Real Time Imaging of Deuterium in a Duplex Stainless Steel Microstructure by Time-of-Flight SIMS
Source: Sci Rep. 2016 Feb 2;6:19929. doi: 10.1038/srep19929 (PMC4735803; doi:10.1038/srep19929)
Supplement: Supplementary Information [file srep19929-s1.pdf]

Real Time Imaging of Deuterium in a Duplex Stainless Steel Microstructure by Time-Of-Flight SIMS

O. Sobol, F. Straub, Th. Wirth, G. Holzlechner, Th. Boellinghaus, W. E. S. Unger

Duplex stainless steel alloying elements:

Table S 1: Duplex Stainless Steel type 2205 (UNS S31803; EN 1.4462) nominal chemical composition[1]

element	C	Cr	Ni	Mo	Mn	N
wt. %	0.03	21.0-23.0	4.5-6.5	2.5-3.5	2.0	0.08-0.20

Analytical characteristics of SIMS

The principal of SIMS is the generation of secondary ions by impact of high energy primary ions which causes a collision cascade within the near surface atomic layers of the investigated area. As a result atomic and molecular fragments are produced and may leave the surface. [2]

There are two modes of SIMS analysis, dynamic and static, both of them were used for investigations of deuterium interactions in steels. In Dynamic SIMS the primary ion flux is aggressively eroding the surface and resulting high sensitivity for trace elements. For static SIMS the secondary ions originate exclusively from non-damaged areas. By the so called “static limit” of 10^{12} primary ions per cm^2 the low primary ion flux density statistically guarantees that every primary ion hits a non-damaged virgin surface. The static mode of operation is achieved by using ToF-SIMS instruments with the capability of parallel mass detection and high transmission of the analyzer.[2] Table S2 presents the experiments where Time-of-Flight Secondary Ion Mass Spectrometry (ToF-SIMS) was used for investigations of hydrogen species in metals. Principally ToF-SIMS, having the parallel mass detection capability at sub-µm lateral resolution, enables both a detailed imaging of the microstructure and an analysis of its chemical composition.[3]. A valid phase identification in the microstructure of DSS is enabled by ToF-SIMS imaging (particularly by using Cr^+, Fe^+, Ni^+ and $CrFe^+$ secondary ion yields as it has been shown in earlier experiments [3]).

Table S 2: Survey of SIMS experiments for elucidation of hydrogen effects

Author	Year	Material	Type of charging	H/D	Type of measurement	Technique	Observations
**Frank et al.** [4]	1982	AISI 302	Cathodic charging	D	Ex-situ depth profiling	Dynamic SIMS	concentration profile in strained and unstrained regions
**Gao et al.** [5]	1994	0.3% C, 1.0% Cr, 1.1% Mn, 0.9% Si steel	Cathodic charging	H	Ex-situ spectra	Dynamic SIMS	distribution of hydrogen in the vicinity of a crack tip under different bending loads
**Oltra et al.** [6]	1994	Duplex stainless steel	tensile test in gaseous or aqueous solution	D	Ex-situ imaging	Dynamic SIMS	deuterium segregation at the crack-tip only in austenitic grains along the crack path
**Oltra et al.** [7]	1996	Duplex stainless steel	tensile test in gaseous or aqueous solution	D	Ex-situ imaging	Dynamic SIMS	deuterium segregation at the crack-tip only in austenitic grains along the crack path
**Takai et al.** [8]	1995	Low-alloyed steel	tensile test in aqueous solution	D	Ex-situ depth profiling and imaging	Dynamic SIMS	Hydrogen and deuterium trapping sites are observable. Accumulation at grain boundaries, in segregation bands and on inclusions
**Takai et al.** [9]	2003	Spheroidal graphite cast iron	Immersion in aqueous solution	H, D	Ex-situ imaging	Dynamic SIMS	visualization of hydrogen desorption from different phases (ferrite, graphite, perlite) depending on the heating temperature
**Kawamoto et al.** [10]	2009	AISI 304	Gas environment	H	Ex-situ depth profiling and imaging around fatigue tip	Dynamic SIMS	fatigue test in gas environment
**Tanaka et al.** [11]	2014	2205 DSS<br>Ni base alloy	Cathodic charging	D	Ex-situ depth profiling and imaging	ToF-SIMS	visualization of hydrogen desorption from different phases (ferrite, austenite)
**Awane et al.** [12]	2014	316L	Gas environment	H	Ex-situ depth profiling	Dynamic SIMS	Time variation of the distribution of hydrogen in the cross-section for diffusion coefficient calculations.

The lateral resolution of the instrument, which is essential especially in the case of this research, is determined by many factors: primary ion beam spot size, mass resolution, signal intensity per pixel, instrument stability (mechanically and electrically), scanning step-width and the dimensions of the collision cascade induced by the primary ion beam in the respective material. In ToF-SIMS, mass resolution and lateral resolution place opposite requirements. Therefore, in each case the best over-all resolution is a compromise between a few factors, most significantly, the primary ion beam.[13] There are different default modes of operating the primary ion source, differentiated by the pulsing cycles. The pulse time defines the mass resolution; the shorter it is – the higher is the mass resolution in the spectra. By "bunching" primary ion pulses (as in high-current bunch mode – HCBU), high mass resolution with narrow peaks can be attained but with a lateral resolution of only 2-5 µm. In order to achieve submicron lateral resolution, the primary ion gun is operated in a non-bunched mode, using longer pulse times of primary ion bombardment. This imaging or burst alignment (BA) mode allows for much better lateral resolution and results in degraded mass resolution.[14] In the BA mode the peaks appear broader and overlapping is inevitable. Therefore in the current research the sample was measured in both modes, acquiring images in a good spatial resolution and allowing analysis of peaks in high mass resolution especially for differentiating secondary ions of the naturally absorbed hydrogen molecularly layers (H_2) and deuterium (D). All images were taken in the same position (ROI) after increasing times of charging with deuterium. After 34 days of charging, when a deuterium concentration was reached enabling to accumulate a good image within a reasonable time, a different ROI was chosen and imaged by both ToF-SIMS and ion induced secondary electron microscopy. Table S3 summarizes the acquisition parameters for all images referred in the main text.

Table S 3: acquisition data for ToF-SIMS images

Charging time [days]	Polarity	Fig. extracted from the spectra	Position on the sample	Acquisition time [h]	Ion dose x 10^{12} [ions·cm^{-2}]	Resolution
**28**	**Negative**	**S1a, S1d**	**A**	**2.25**	**0.56**	**128X128**
**34**	**Positive**	**2a**	**A**	**4.5**	**1.6**	**128X128**
**34**	**Negative**	**S1b, S1e, 2b, 2c, 2d**	**A**	**4.5**	**1.6**	**128X128**
**37**	**Positive**	**3a**	**B**	**4.5**	**1.6**	**256X256**
**37**	**Negative**	**S1c, S1f, 3b**	**B**	**4.5**	**1.6**	**256X256**
**37**		**3c, Ion induced secondary electrons**	**B**			**256X256**

In both dynamic and static SIMS, surface spectra can be obtained either in the positive or in the negative analysis mode, gaining analytical and spatial information as a function of sensitivity for a specific element in every analysis mode. In the current investigation both negative and positive data were obtained in sequential experiments by switching the polarity of the whole mass spectrometer. The negative mode was used for obtaining D^-, OD^-, CN^-, CN_2HD^- signals and the positive mode for distinguishing the chemical composition of ferrite and austenite.

The first step of the experiment was to image the finely polished DSS surface of the sample before charging by ToF-SIMS in the positive mode. A microstructurally representative region of 100X100μm^2 was selected to identify the austenitic and the ferritic phase by the detection of Cr^+, Ni^+, Mn^+ and Fe^+ secondary ions; first with instrument settings delivering high mass resolution and thereafter with settings for high lateral resolution[3]. Subsequently, negative secondary ion images were acquired at increasing times of charging DSS with deuterium over a period up to 37 days. Image data were treated by PCA. This leads to appropriate contrast and details. Figures 2 and 3 show score plots for different principle components derived from positive or negative secondary ion image data sets 34 days after starting the deuterium permeation cell. D^- and PC1 score images taken at 28 days, the breakthrough time of deuterium, are shown in the following for the sake of comparison.

PCA and data pre-processing

In the current study, by increasing charging times, enhanced deuterium count rates are observed when investigating the steel surface. For a specific mass, solely, the measured secondary ion intensities in the normalized images is very low and hence a clear correlation of the lateral distribution of different species within the DSS steel microstructure is difficult. The PCA method assisted here for identifying and

enhancing the contrast between chemically different regions by looking at the main variances within a data set of secondary ions. PCA uses a matrix containing the measured mass spectra in its rows and the individual mass channels in its columns. Calculated from the covariance of the matrix PCA generates new matrices containing, among others, the scores and the loadings that together represent a concise summary of the original data.[15,16]

Subsequently to all SIMS measurements principle component analysis was used to identify trends in the collected data set of secondary ions. Within this process, identification of key co-variances between the mass spectra features enables enhancement of the contrast. Prior to principle component analysis the raw data of secondary ions were pre-processed by normalizing to the individual total intensity of the mass spectra, Poisson scaling and mean centering. Loadings plots of all PCA processed images are shown in figure S2a-d. Figure S2a refers to figures 2a and 3a in the main text. Figure S2b refers to figure 2b and 3b, figure S2c-d refer to figure 2c-d, respectively. As mentioned, the reasons for selecting specific peaks in the spectrum for applying PCA come from two reasons:

1. For extracting only useful information from the data and removing noise peaks
2. High overlapping in high masses due to the formation hydrides and hydroxide of hydrogen and deuterium in addition to the natural metal isotopes.

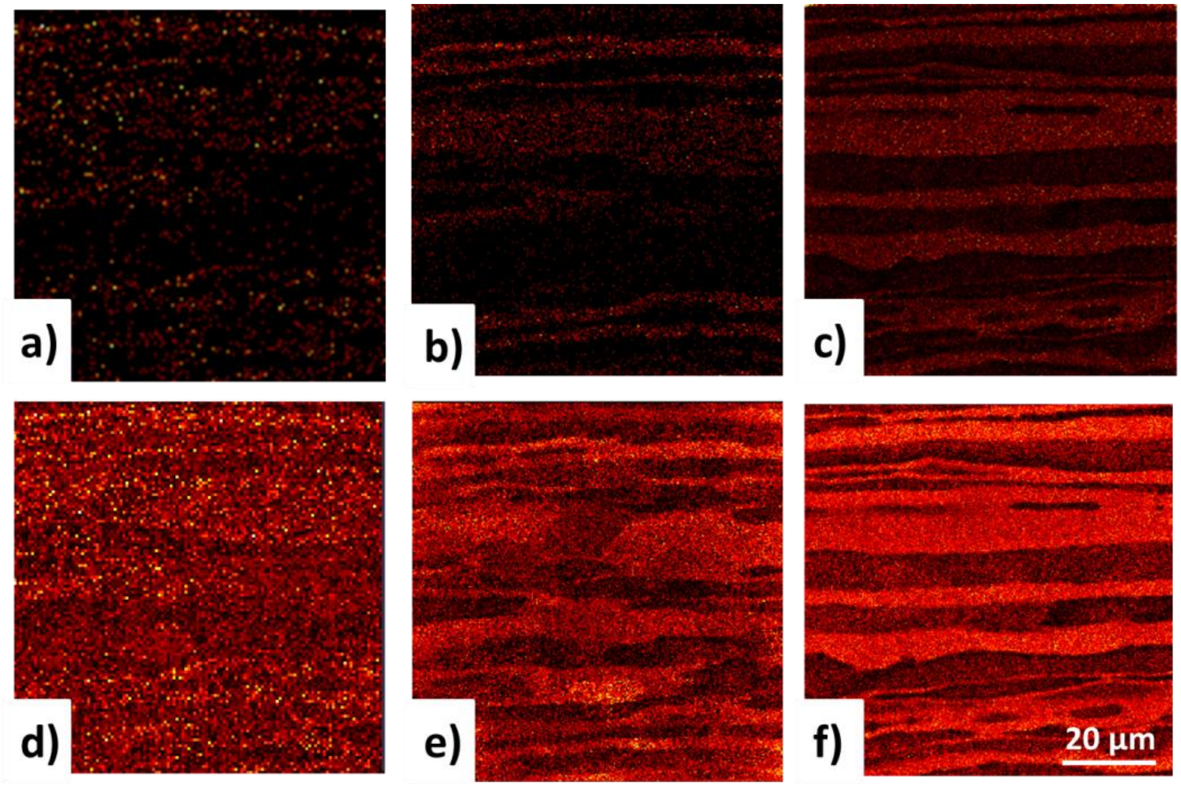

Supplementary figure 1: Time resolved deuterium accumulations in the analyzed surface and the comparison of the D^- ions raw data images to PCA contrast enhanced images given in the main text. a-c) normalized raw data images of only the D^- signal taken after 28, 34 and 37 days, respectively; d-f) PCA enhanced images of images a-c, respectively. All images were taken from a 100x100 μm^2 area.

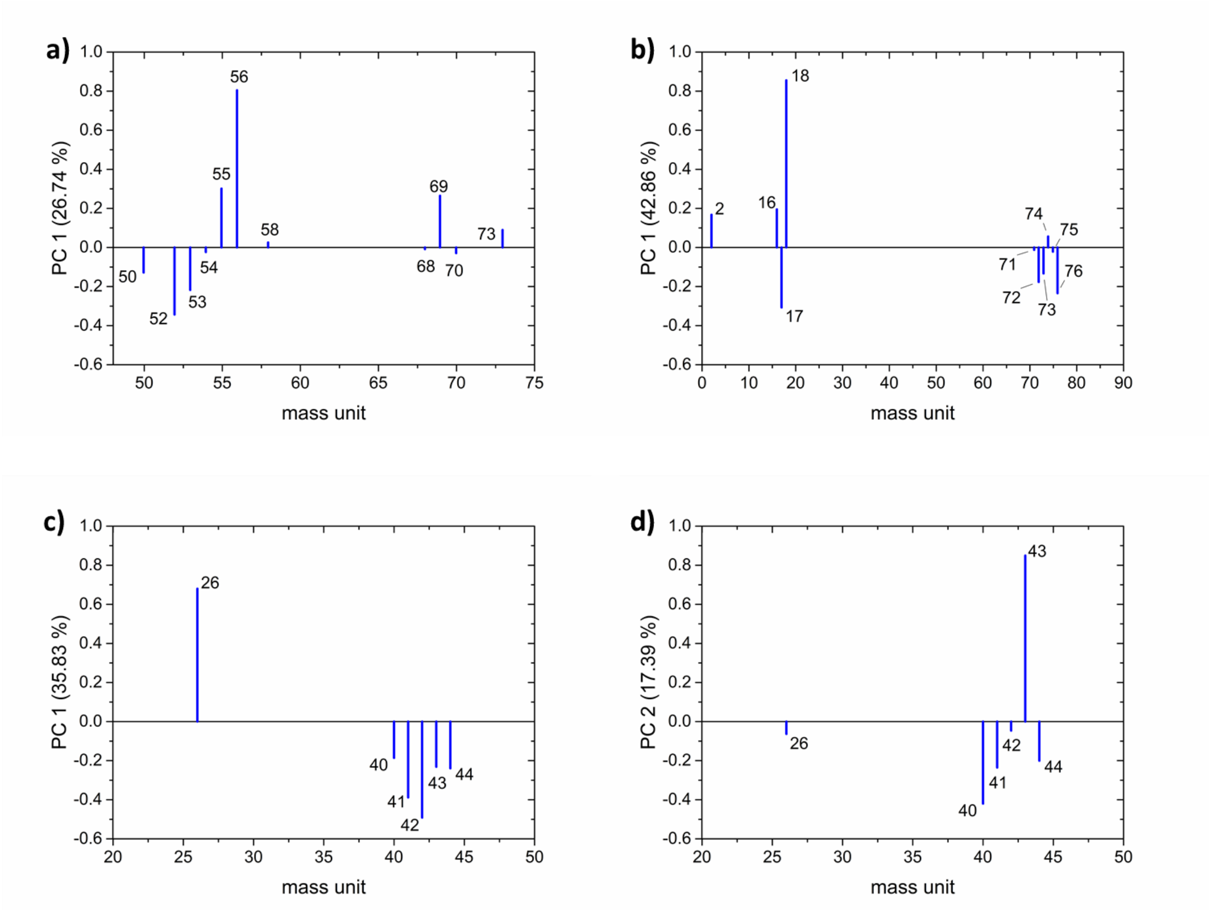

Supplementary figure 2: Loading plots from PCA corresponding a) to the scores in Fig 2a and 3a; b) to the scores in Fig 2b and 3b; c) to the scores in Fig 2c; d) to the scores in Fig 2d.

Table S 4: Measured m/z values shown in the loading plots in figure S2a-d with their assignments

Observed m/z	Suggested assignment (positive SIMS mode)	Suggested assignment (negative SIMS mode)
2		D
16		O
17		OH
18		OD
26		CN
40		C_2O
41		C_2OH
42		CNO
43		CN_2HD
44		$NOCH_2$
50	^{50}Cr	
52	^{52}Cr	
53	^{53}Cr	
54	^{54}Cr	
55	Mn	
56	Fe	
58	Ni	
68	FeC	
69	FeCH, $CrNH_3$	
70	$FeCH_2$, $^{53}CrNH_3$	NiC
71		$^{54}CrOH$
72		FeO
73	$FeNH_3$, FeOH	FeOH
74		FeOD
75		NiOH, $FeOH_3$
76		$NiOH_2$

References:

1 Alvares-Armas, I. Duplex Stainless Steels: Brief History and Some Recent Alloys. *Recent Patents on Mechanical Engineering* 1, 51-57 (2008).

2 Unger, W. E. S. & Hodoroaba, V. D. *Surface Chemical Analysis at the Micro-and NanoScale*. 301-322 (Springer, 2013).

3 Straub, F. *et al.* Imaging the microstructure of duplex stainless steel samples with TOF-SIMS. *Surface and Interface Analysis* 42, 739-742, doi:10.1002/sia.3385 (2010).

4 Frank, R. C., Baker, J. E. & Altstetter, C. J. A Sims Study of the Diffusion and Trapping of Deuterium in 302 Stainless-Steel. *Metallurgical Transactions a-Physical Metallurgy and Materials Science* 13, 581-584, doi:10.1007/bf02644422 (1982).

5 Gao, H., Cao, W., Fang, C. & Delosrios, E. R. Analysis of Crack-Tip Hydrogen Distribution under I/Ii Mixed-Mode Loads. *Fatigue Fract. Eng. Mater. Struct.* 17, 1213-1220, doi:10.1111/j.1460-2695.1994.tb01410.x (1994).

6 Oltra, R. & Bouillot, C. Experimental investigation of the role of hydrogen in stress corrosion cracking of duplex stainless steels. *Hydrogen Transport and Cracking in Metals*, 17-26 (1994).

7 Oltra, R., Bouillot, C. & Magnin, T. Localized hydrogen cracking in the austenitic phase of a duplex stainless steel. *Scr. Mater.* 35, 1101-1105, doi:10.1016/1359-6462(96)00293-x (1996).

8 Takai, K., Seki, J. & Homma, Y. Observation of Trapping Sites of Hydrogen and Deuterium in High-Strength Steels by Using Secondary-Ion Mass-Spectrometry. *Materials Transactions Jim* 36, 1134-1139 (1995).

9 Takai, K., Chiba, Y., Noguchi, K. & Nozue, A. Visualization of the hydrogen desorption process from ferrite, pearlite, and graphite by secondary ion mass spectrometry. *Metallurgical and Materials Transactions a-Physical Metallurgy and Materials Science* 33, 2659-2665, doi:10.1007/s11661-002-0387-8 (2002).

10 Kawamoto, K. *et al.* Investigation of Local Hydrogen Distribution Around Fatigue Crack Tip of a Type 304 Stainless Steel with Secondary Ion Mass Spectrometry and Hydrogen Micro-Print Technique. *Journal of Solid Mechanics and Materials Engineering* 3, 898-909 (2009).

11 Tanaka, T., Kawakami, K. & Hayashi, S.-i. Visualization of deuterium flux and grain boundary diffusion in duplex stainless steel and Fe-30 % Ni alloy, using secondary ion mass spectrometry equipped with a Ga focused ion beam. *Journal of Materials Science* 49, 3928-3935, doi:10.1007/s10853-013-7956-7 (2014).

12 Awane, T., Fukushima, Y., Matsuo, T., Murakami, Y. & Miwa, S. Highly Sensitive Secondary Ion Mass Spectrometric Analysis of Time Variation of Hydrogen Spatial Distribution in Austenitic Stainless Steel at Room Temperature in Vacuum. *International Journal of Hydrogen Energy* 39, 1164-1172, doi:10.1016/j.ijhydene.2013.10.116 (2014).

13 Vickerman, J. C. & Briggs, D. 789 (IM Publications; SurfaceSpectra Ltd., Manchester, UK, 2001).

14 Sodhi, R. N. S. Time-of-flight secondary ion mass spectrometry (TOF-SIMS): versatility in chemical and imaging surface analysis. *Analyst* 129, 483-487, doi:10.1039/b402607c (2004).

15 Lee, J., Gilmore, I., Fletcher, I. & Seah, M. Multivariate image analysis strategies for ToF-SIMS images with topography. *Surface and Interface Analysis* 41, 653-665 (2009).

16 Graham, D. J. & Castner, D. G. Multivariate analysis of ToF-SIMS data from multicomponent systems: the why, when, and how. *Biointerphases* 7, 10, doi:10.1007/s13758-012-0049-3 (2012).
